# Natural allelic variation of the *AZI1* gene controls root growth under zinc-limiting condition

**DOI:** 10.1371/journal.pgen.1007304

**Published:** 2018-04-02

**Authors:** Nadia Bouain, Santosh B. Satbhai, Arthur Korte, Chorpet Saenchai, Guilhem Desbrosses, Pierre Berthomieu, Wolfgang Busch, Hatem Rouached

**Affiliations:** 1 BPMP, Univ Montpellier, CNRS, INRA, SupAgro, Montpellier, France; 2 Gregor Mendel Institute (GMI), Austrian Academy of Sciences, Vienna Biocenter (VBC), Vienna, Austria; 3 Plant Molecular and Cellular Biology Laboratory, Salk Institute for Biological Studies, La Jolla, United States of America; 4 Evolutionary Genomics Center for Computational and Theoretical Biology (CCTB), University Würzburg, Würzburg, Germany; John Innes Centre, UNITED KINGDOM

## Abstract

Zinc is an essential micronutrient for all living organisms and is involved in a plethora of processes including growth and development, and immunity. However, it is unknown if there is a common genetic and molecular basis underlying multiple facets of zinc function. Here we used natural variation in *Arabidopsis thaliana* to study the role of zinc in regulating growth. We identify allelic variation of the systemic immunity gene *AZI1* as a key for determining root growth responses to low zinc conditions. We further demonstrate that this gene is important for modulating primary root length depending on the zinc and defence status. Finally, we show that the interaction of the immunity signal azelaic acid and zinc level to regulate root growth is conserved in rice. This work demonstrates that there is a common genetic and molecular basis for multiple zinc dependent processes and that nutrient cues can determine the balance of growth and immune responses in plants.

## Author summary

Plants have evolved mechanisms to cope with complex environments in which resources as well as potential threats are fluctuating. Thereby, plants modulate their growth based on multiple cues from the environment. In this study, by exploring natural genetic variation in Arabidopsis to study the role of zinc in regulating primary root length, we find a major locus governing this is the *AZELAIC ACID INDUCED* (*AZI1*) locus, previously known to be involved in systemic acquired resistance. We then showed that regulatory variation at *AZI1* contributes significantly to this natural variation. Importantly, the known AZI1 function led us to show that there is an interaction of zinc deficiency and the defence pathway. While the studies of the roles of the defence signal AzA and AZI1 had been restricted to the aboveground tissues, we clearly showed an important role of this pathway in the root, which is zinc-dependant. Our observations regarding the interaction of zinc and AzA-dependent defence pathways on root growth are not a fluke of evolution, but they are evolutionary conserved between dicots and monocots. Taken together, these results will serve as a basis to design new strategies for improvement agricultural crop species able to modulate growth and defence.

## Introduction

Zinc (Zn) is an essential micronutrient for humans, animals, and plants [1]. It is of particular importance for the function of numerous metalloenzymes that are involved in a plethora of processes such as energy metabolism, nucleic acid and protein synthesis, and protein catabolism [2]. These key biological processes can be adversely altered in situations in which Zn availability is limited. Low Zn manifests itself at physiological and molecular levels, and can cause deleterious effects such as growth retardation and malfunction of immune responses. Recent studies in mammalian systems have shifted the focus on the role of Zn from simply a nutrient to a signalling molecule that fine-tunes intracellular signalling events (for an overview see [3]) and an important player in nutritional immunity [4]. In particular, in the role of Zn for host defence, a complex role is emerging. On one hand, Zn is actively depleted from infection sites restricting the ability of the pathogen to proliferate [5, 6] and on the other hand high Zn levels are generated by the host that contribute to kill the pathogen [7]. However, despite its fundamental importance, it remains unclear whether there is a common molecular basis for these multiple functions involving Zn and whether the signalling and immune related functions of Zn are also relevant for plants.

Plants are the first link in the chain of human Zn nutrition and therefore studying Zn related processes in plants is of particular relevance. Land plants acquire Zn at the root–soil interface and multiple processes are crucial for efficient Zn acquisition. While Zn transport is clearly very important for Zn acquisition and homeostasis [8], other processes also play pronounced roles for efficient growth under Zn limited conditions. For instance, when responses of two lines of rice that displayed a contrasting tolerance to -Zn were quantified, these lines didn’t differ in Zn-transporter activity but mostly in their maintenance of root growth and the exudation rates of organic acids [9]. A similar correlation of increased root growth and increased tolerance to –Zn conditions had also been described in wheat [10]. Overall, this indicated that Zn levels in the environment are perceived by the plant and lead to distinct changes in root growth that might be important for adaptive responses to low Zn conditions. Nevertheless, so far, neither a role for Zn as signal, nor the genetic and molecular bases of root growth changes upon -Zn conditions in plants has been clearly established yet.

One important biotic stress response process that Zn and its availability has been shown to be involved in animals [11] as well as in plants is disease resistance [12]. One gene that is involved in the response to biotic and abiotic stresses in *Arabidopsis thaliana* is *AZI1* (*AZELAIC ACID INDUCED 1*, At4g12470) [13, 14]. It encodes for a lipid transfer protein (LTP)-like protein and belongs to the *EARLY ARABIDOPSIS ALUMINIUM-INDUCED GENE1* (*EARLI1*) gene subfamily [15]. *AZI1* was named after its unique response to the systemically active compound azelaic acid (AzA, a nine-carbon dicarboxylic acid) [16]. In the Arabidopsis genome, *AZI1* clusters in a tandem array on chromosome 4 with three other *EARLI1*-type genes, namely At4g12480 (*EARLI1*), AT4G12490 (*ELHYPRP2 (EARLI1-LIKE HYBRID PROLINE-RICH PROTEIN 2*) and AT4G12500. Among these genes, the role of *AZI1* for long-distance signals related to systemic acquired resistance (SAR) is the best documented so far. In addition to *AZI1*, SAR involves an important hormone, namely salicylic acid (SA). SA accumulates upon pathogen attack [17], and leads to the induction of *PATHOGENESIS-RELATED GENE 1* (*PR1*, SA marker gene) [18]. In *Arabidopsis thaliana*, mutation of *AZI1* causes a specific loss of systemic immunity triggered by pathogens [16]. Beyond its role in biotic stress responses, the involvement of *AZI1* in response to abiotic stress, such as the regulation of seedling growth under salt stress, was demonstrated [14]. But, whether *AZI1* is involved in the response to nutrient levels that potentially affect the plants capability to defend itself or the capability for pathogens for infection (e.g. low Zn), remains unexplored.

Here, we study the genetic basis of low exogenous Zn levels on primary root length by exploring natural genetic variation. We find that there is heritable natural variation of root length responses to low Zn and that natural allelic variation of the immune gene *AZI1* determines a significant proportion of this response. We further reveal an intriguing evolutionarily conserved interaction between exogenous Zn levels and AzA dependent defence pathways to regulate primary root elongation.

## Results

### The *AZI1* gene is involved in the regulation of root length to low Zn conditions

To identify genetic components that regulate plant growth upon low Zn (-Zn) conditions, we determined the length of the primary root of 231 genetically diverse natural accessions of *Arabidopsis thaliana* (S1A Fig) from the RegMap population [19] grown on +Zn and -Zn medium over 7 days (S1 and S2 Tables). Importantly, while still being correlated, the primary root length of the vast majority of accessions clearly differed in +Zn and –Zn conditions (S1B and S1C Fig). To assess whether these root length responses were specifically due to the -Zn treatments, we determined mRNA levels of four Zn-deficiency responsive marker genes *ZIP3*, *ZIP5*, *ZIP12* [20] and *PHO1;H3* [21] in the Col-0 accession under our screening conditions. All of these four genes were significantly up-regulated in –Zn conditions (S2 Fig), demonstrating that the plants sensed and responded to the -Zn conditions.

**Fig 1:**
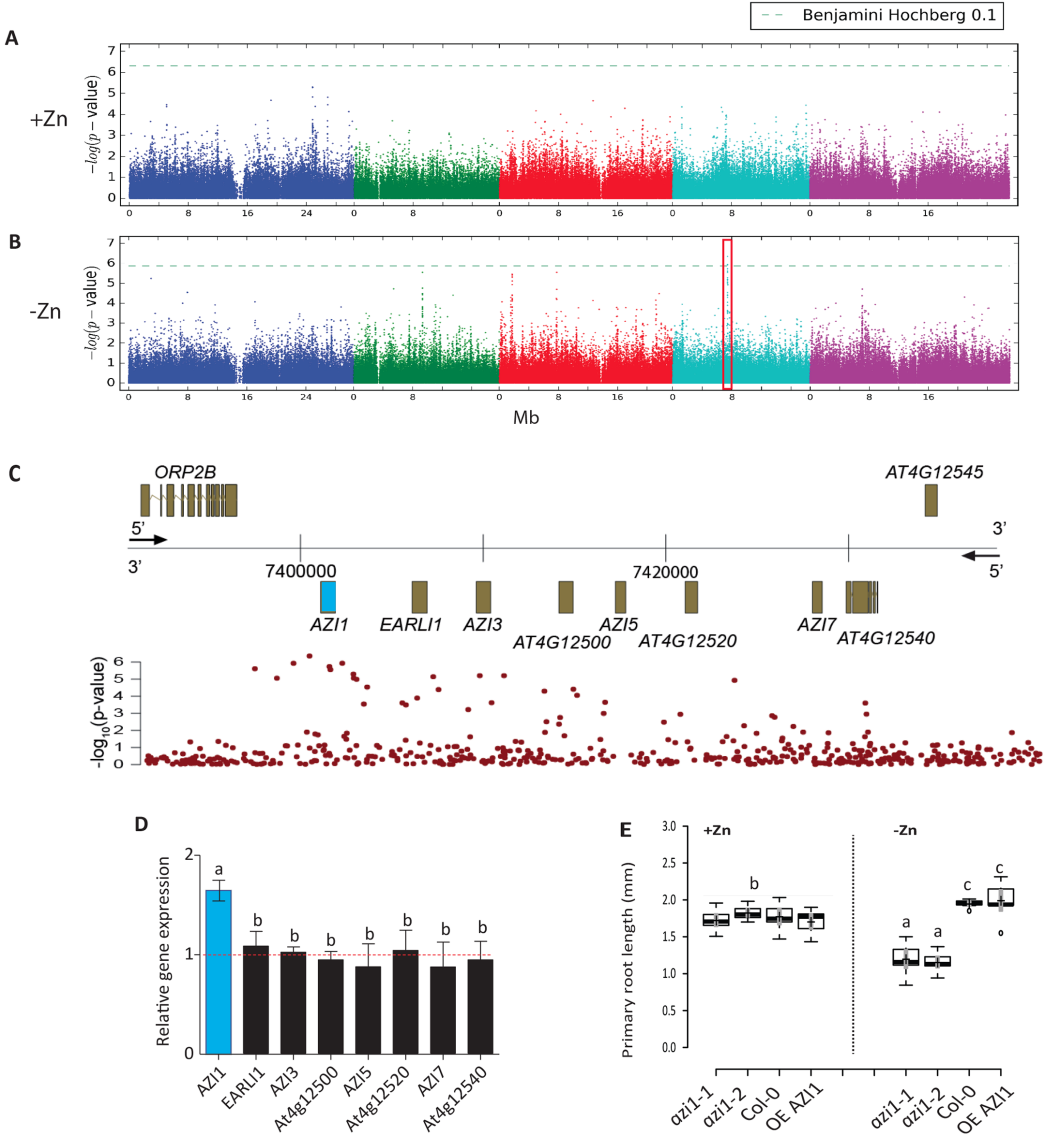
AZI1 controls root growth under zinc limiting conditions. GWAS for mean root length based on a set of 231 *A*. *thaliana* accessions grown under (A) zinc sufficiency (+Zn) or (B) low zinc (-Zn) (day 2). The chromosomes are represented in different colors. The horizontal dash-dot line corresponds to a FDR of 10% after Benjamini–Hochberg–Yekutieli correction. The red box indicates the significant association. (C) Genomic region around *AZI1* locus (highlighted in blue). X-axis: genomic position. Y-axis: upper panel: Gene models. Lower panel: LOD score from GWAS depicted in (B). (D) Expression changes (fold change) of *AZI1*, *EARLI1*, AZI3, At4g12500, AZI5, At4g12520, *AZI7* and At4g12540 in Col-0 grown in –Zn conditions compared to Col-0 plants grown under +Zn conditions. Every data point was obtained from the analysis of roots collected from a pool of ten plants. Error bars correspond to s.d.; three biological repeats. The *Ubiquitin* gene was used as an internal reference. (E) Average primary root length (Day 2) of wild-type plants (Col-0 genotype), *azi1* mutant and overexpressor line 35S::*AZI1* (OE *AZI1*) plants grown under +Zn or –Zn respectively. Crosses show sample mean; center lines show sample medians; box limits indicate the 25th and 75th percentiles as determined by R software; whiskers extend 1.5 times the interquartile range from the 25th and 75th percentiles. Outliers are represented by dots. Shaded central region show confidence intervals of means. This graph was generated by BoxPlotR: a web-tool for generation of box plots [15]. Experiments were independently repeated three times, and data are represented as mean ± s.d. n = 10. Letters a, b and c indicate significantly different values at p <0.05 determined by one-way ANOVA and Tukey HSD.

In the panel of screened accessions, we observed broad phenotypic variation for root length (S3 Fig) that was highly heritable (broad sense heritability (H^2^) ranging [22] from 0.36 to 0.44 for –Zn, and from 0.42 to 0.48 for +Zn) (S3 Table). Our analysis revealed that nearly 20% of the respective phenotypic variance is accounted for by genotype by environment (G X E) interactions for all days (S4 Table). We then conducted Genome Wide Association Studies (GWASs) using the AMM method that corrects for population structure confounding [23], to identify loci that were associated specifically with root length under -Zn (Fig 1A and 1B, S4 Fig). No association was observed under +Zn (S5 Fig). We then corrected the association *P*-values for all SNPs for multiple testing using the Benjamini-Hochberg-Yekutieli method [24]. Due to the limited power of our association study and the potentially high false negative rate due to population structure correction, and because we aimed to test any major emerging candidate experimentally, we selected a relatively non-conservative 10% false discovery rate (FDR) as our threshold for significant association (we thus expect that 10% of the significant SNPs are false positives). Using this criterion, we identified two chromosomal regions associated with root length in –Zn conditions. On chromosome 2, the significant peak (*P*-value = 3.27*10^−7^; FDR ≈ 7%) was located in a region with a cluster of similar genes encoding a Cysteine/Histidine-rich C1 domain family (At2g21810). It was detected on the last day of the time course (day 7). These proteins require Zn ions for their function [25] (pfam, PF00130). On chromosome 4, the significant peak (*P*-value = 4.40*10^−7^; FDR ≈ 6%) was located in in a region that contains the lipid transfer protein (LTP)-like *AZI1* (At4g12470) gene and the 7 additional genes encoding for lipid transfer proteins as a cluster (Fig 1B and 1C). This peak was already detected early in the duration of our time course (day 2) suggesting that this locus was relevant for regulating early primary root length in response to limitations in external Zn rather than being a consequence of low internal Zn levels. As *AZI1* itself was known for being involved in signalling: it mediates azelaic-acid-induced systemic immunity [16]; we hypothesized that *AZI1* was involved in mediating crosstalk between nutrient and immunity signals. However, as the GWAS peak spanned multiple genes, we first tested whether the best candidate in this region was indeed *AZI1*. For this, we assessed all 8 genes in the genomic region surrounding the association peak. Of these 8 genes, only *AZI1* showed significant transcript level alteration in response to -Zn in Col-0 (Fig 1D), suggesting that it was involved in –Zn dependent root growth regulation. To test this further, we determined the root lengths of Arabidopsis Col-0 (WT), two *azi1* (T-DNA) mutant lines (*azi1-1* and *azi1-2*) and an *AZI1* overexpressing (OE *AZI1*) line (35S::*AZI1*) grown in +Zn or -Zn conditions over 7 days (S6 and S7 Figs). In presence of Zn, no significant differences in root length could be observed between *azi1* mutant lines and wild-type plants (day2, Fig 1E; 7 days, S6A Fig). Grown under -Zn, the root length of *azi1* was significantly shorter than Col-0 and 35S::*AZI1* plants (day 2, Fig 1E; 7 days, S6B Fig). Starting from day 5 onwards, roots of *azi1* mutant lines were still significantly shorter and roots of 35S::*AZI1* plants become significantly longer than Col-0 roots (S6 Fig, day 5; S7A and S7B Fig), which suggests that the expression level of *AZI1* is involved in controlling this trait (root length) (S6C Fig). The effect of Zn limitation was only visible on primary roots length. Day 5 was therefore chosen as time point for further analysis. To assess whether this is a function of *AZI1* that is common to other micronutrients, we grew the same set of lines under low iron (-Fe) conditions (S7C Fig). There, no significant root length difference could be detected for the four tested lines, which indicates that growth responses to comparable nutrient limitations are not dependent on *AZI1* and supports the notion of a rather specific *AZI1* dependent response to Zn.

While it has been shown that *AZI1* transcripts accumulate in the aboveground tissues [16], they can be detected in roots as well (S8 Fig, [26]). Therefore, in order to assess whether the effects observed on root length in –Zn are associated with a systemic role of *AZI1* or its local expression in roots, we expressed *AZI1* under the control of the promoter of the zinc transporter *ZIP1*, which is known to be predominantly expressed in roots specifically under Zn deficiency [27]. In line with these results [27], *AZI1* transcript was only detected in the roots of Zn-deficient pZIP1::*AZI1* seedling (S9A Fig). Consistent with a local role of *AZI1* in roots, 2 independent single-insertion pZIP1::*AZI1/azi1* lines displayed longer roots than Col-0 under –Zn conditions, while the roots of *azi1* mutant lines were significantly shorter than Col-0 (S9B and S9C Fig). Taken together, these data show that *AZI1*, previously described as a key component of plant systemic immunity involved in priming defence [16, 28], modulates primary root length in a Zn level dependent manner.

### Natural allelic variation of *AZI1* determines primary root length under zinc limiting conditions

**Fig 2:**
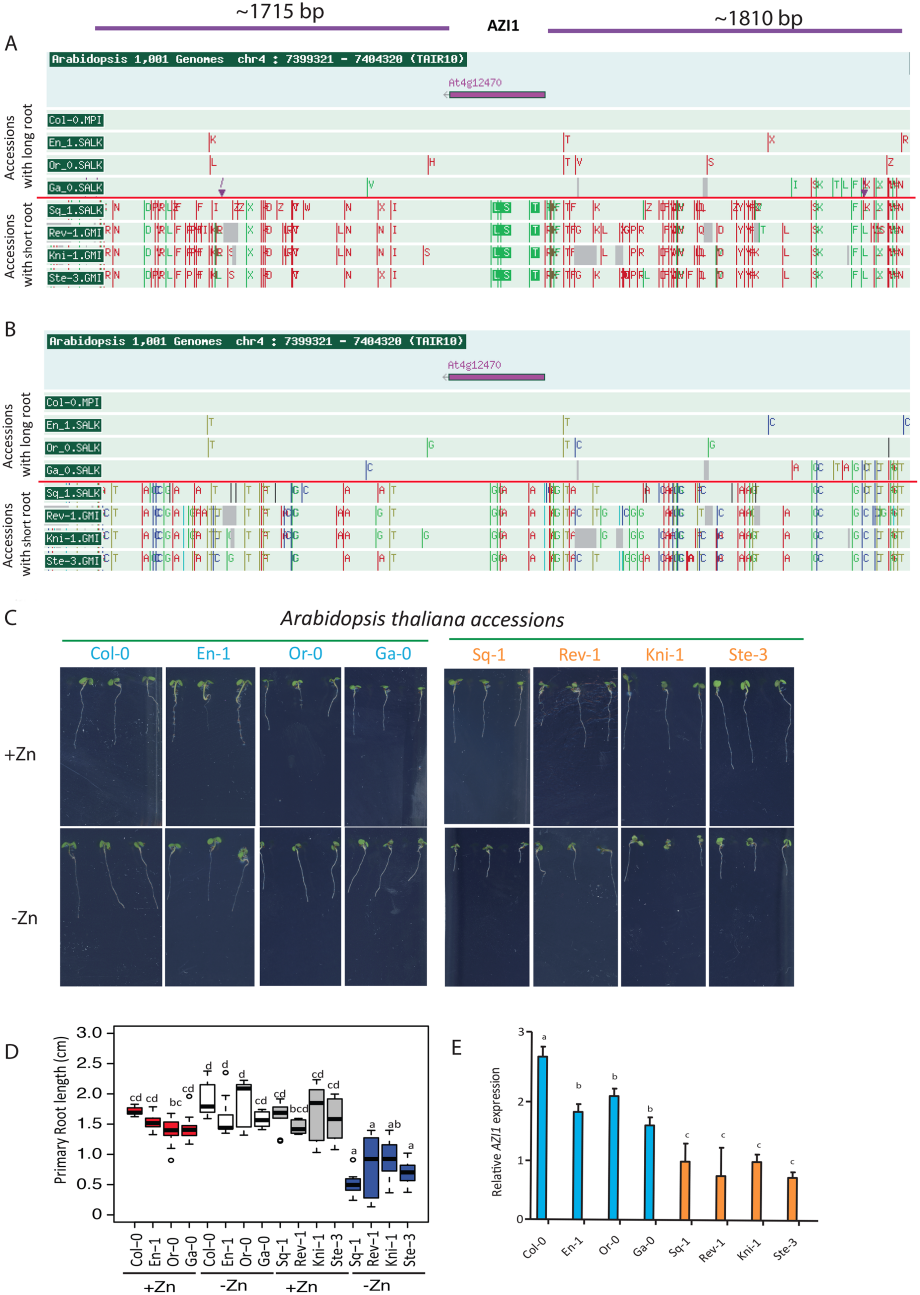
Polymorphism patterns around the *AZI1* locus in extreme accessions. (A, B) Gene models and SNP polymorphisms among representative extreme accessions (4 accessions with short root phenotype in -Zn and 4 accessions with long root phenotype in -Zn) for the genomic region surrounding the *AZI1* gene (~1810 bp upstream and ~1715 bp downstream the start codon of the *AZI1)*. (A) Amino acid changes around *AZI1* (At4g12470) locus. (B) SNPs around *AZI1* locus; Synonymous amino acid: green line, non- synonymous amino acid: red line. Only genomes that were available in the SALK 1001 genomes browser (http://signal.salk.edu/atg1001/3.0/gebrowser.php) as of August 2016 were considered. (C) Representative images of contrasting PRG phenotype (day 5) of eight Arabidopsis thaliana accessions grown in +Zn or –Zn conditions. (D) Average primary root length (Day 5) of these eight accessions grown in –Zn and +Zn conditions. Experiments were independently repeated three times. Crosses show sample mean; center lines show sample medians; box limits indicate the 25th and 75th percentiles as determined by R software; whiskers extend 1.5 times the interquartile range from the 25th and 75th percentiles. Outliers are represented by dots. Shaded central region show confidence intervals of means. This graph was generated by BoxPlotR: a web-tool for generation of box plots. (E) Transcript accumulation of *AZI1* in roots of these eight accessions grown for 5 days in –Zn conditions compared to +Zn conditions. Arabidopsis *Ubiquitin* gene was used as an internal reference. The data are given as means ± s.d. n = 10.

**Fig 3:**
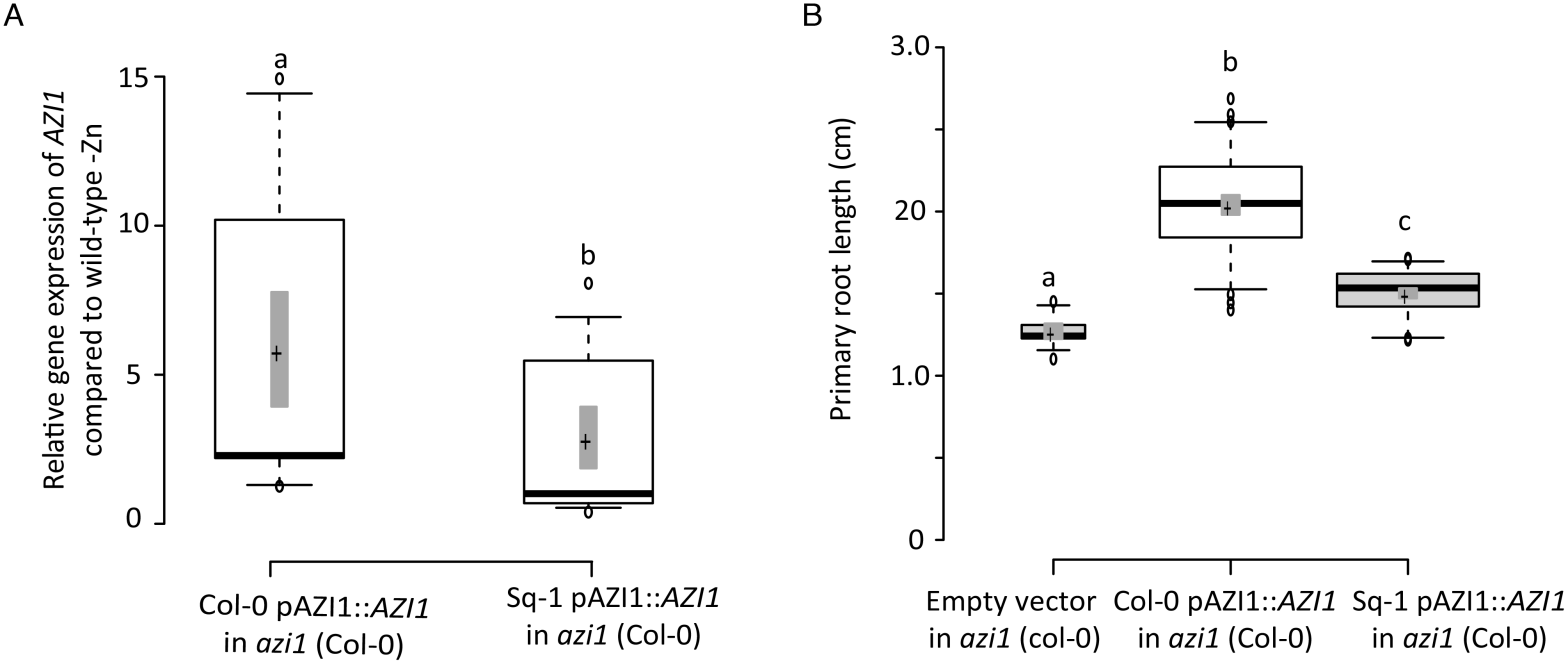
Natural allelic variation of the *AZI1* locus underlies phenotypic variation of root length in zinc limiting conditions. (A) Transcript level of *AZI1* in *azi1* lines complemented with pAZI1:*AZI* from either Col-0 or Sq-1 on –Zn conditions and shown as relative to +Zn condition (5 days). Five independent T3 lines were considered for this analysis. Relative expression was quantified in three biological replicates using RT-qPCR. The Ubiquitin gene was used as an internal reference. (B) Primary root length of *azi1* lines complemented with pAZI1:*AZI1* from either Col-0 (n = 50), Sq-1 (n = 50) or the empty vector (n = 10) on –Zn conditions (5 days). For each genotype, three repeats each containing five independent T3 lines. Box plots show analysis of the relative gene expression of *AZI1* (A) and primary root length (B) in of *azi1* lines complemented with pAZI1:*AZI* from either Col-0, Sq-1 or the empty vector on –Zn conditions. Crosses show sample mean; center lines show sample medians; box limits indicate the 25th and 75th percentiles as determined by R software; whiskers extend 1.5 times the interquartile range from the 25th and 75th percentiles. Outliers are represented by dots. Shaded central region show confidence intervals of means. This graph was generated by BoxPlotR: a web-tool for generation of box plots. Letters a, b and c indicate significantly different values at p <0.05 determined by one-way ANOVA and Tukey HSD.

While we had shown that *AZI1* was involved in modulating primary root length in a Zn level dependent manner, this was not proof that the allelic variation of *AZI1* is causal for the observed root length differences under –Zn. We therefore set out to test this. Sequence analysis of the *AZI1* genomic region (promoter and coding region) showed multiple polymorphisms in the regulatory region (S5 Table) as well as synonymous changes in the coding region that were consistently different between contrasting groups of accessions with either long or short roots on –Zn (Fig 2A and 2B, S10 Fig, S1, S2 and S6 Tables). Consistent with causal regulatory polymorphisms, *AZI1* expression was significantly higher upon –Zn in accessions with longer roots (Fig 2C, 2D and 2E). For further analysis, we focussed on two contrasting accessions, Col-0 and Sq-1, which were among the most contrasting accessions regarding their root length on –Zn (S1 and S2 Tables) and each displayed the variant of the marker SNP that was associated with long and short roots on –Zn respectively. To then experimentally test whether the difference in *AZI1* expression level was due to the natural allelic variation and whether the allelic variation was also causal for the longer roots, we transformed the *azi1* mutant (Col-0 background) with constructs containing 1.6kbp of the promoter and the coding region from either Col-0 (long roots in -Zn) or Sq-1 (short roots in -Zn), and an empty vector (control). In five independent homozygous single insertion lines complemented with the Col-0 pAZI1:*AZI1* the expression level of *AZI1* under –Zn was significantly higher (P<0.01) than that in plants transformed with the Sq-1 pAZI1:*AZI1* construct (Fig 3A). Consequently, we tested these T3 lines for root length differences under –Zn and +Zn. Consistent with the hypothesis that our *AZI1* variants determine root growth specifically under –Zn, no difference in term of root length was observed between the T3 lines grown on +Zn (S11 Fig), while under *-Zn*, we observed significantly longer roots (P< 0.05) in the Col-0 pAZI1:*AZI1* plants compared to Sq-1 pAZI1:*AZI1* plants or *azi1* plants transformed with the empty vector (Fig 3B). Taken together, these data demonstrate that allelic variation at the *AZI1* locus can cause variation of *AZI1* expression levels and at the same time leads to variation of primary root length under -Zn. Moreover, as there were multiple polymorphisms in the regulatory region of the *AZI1* gene (Col-0 and Sq-1 accessions, S12A Fig), and only synonymous changes in the coding sequence in these constructs (S12B Fig), we can rule out that the observed effect is due to changes in the protein sequence of AZI1. Therefore, our data suggest that the differences caused by the two *AZI1* alleles are due to regulatory elements or posttranscriptional regulation such as RNA stability. We note, that we cannot completely exclude the additional involvement of other genes in the associated region in contributing to this response.

### Azelaic acid inhibits Arabidopsis primary root length in a Zn-dependent manner

**Fig 4:**
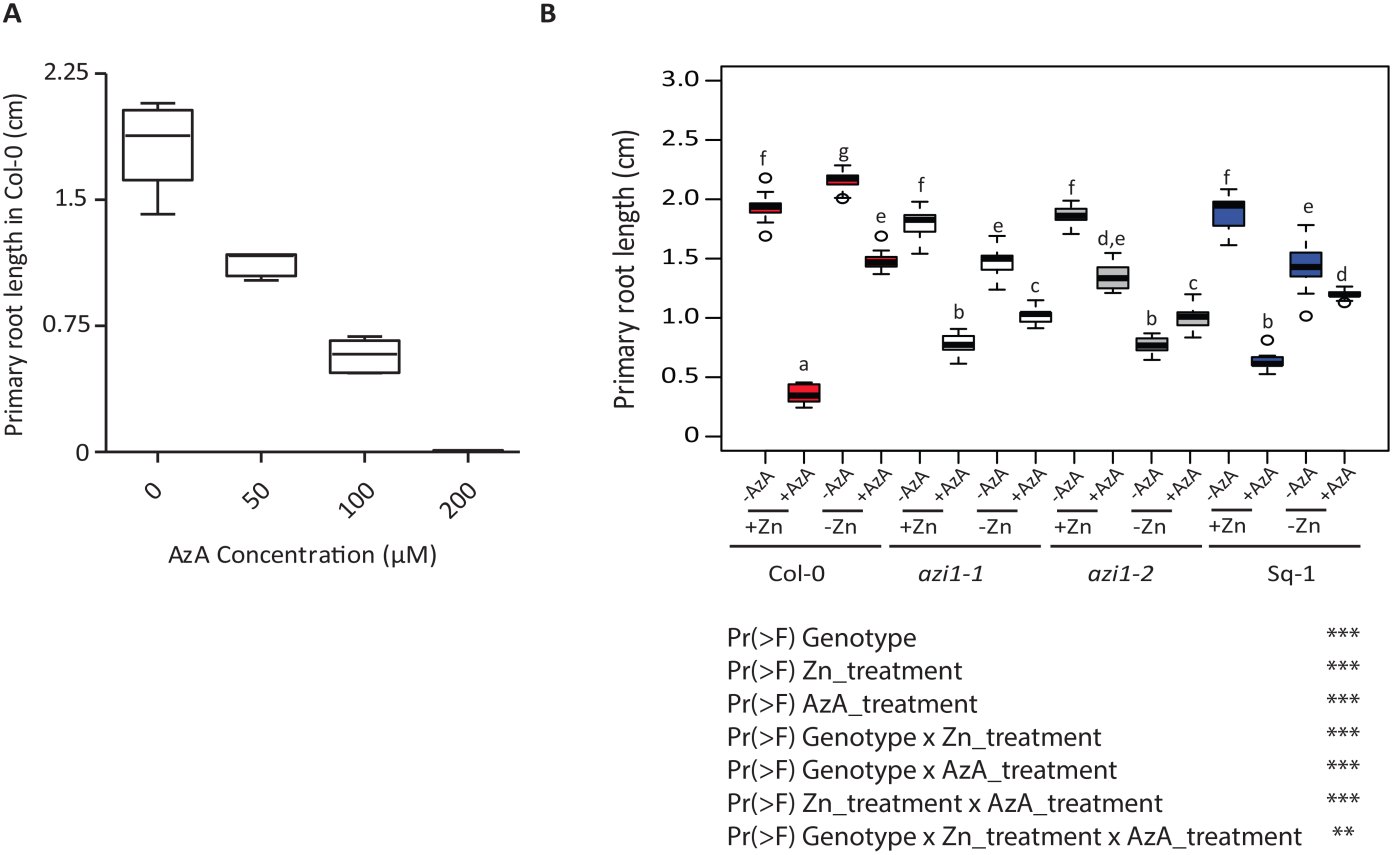
Zinc level and Azelaic acid interact to regulate early root length in an *AZI1* dependent fashion. (A) Root lengths of wild-type seedlings treated with different AzA concentrations on +Zn for five days. Box and whisker plots were generated using Prism (Graphpad), with the box represents the 25th to 75th percentiles and the whiskers reaching to the lowest and highest values. The line in the box shows the median. (B) Root lengths of 5-day-old Col-0, Sq-1 and *azi1 (azi1-1*, *azi1-2)* seedlings treated with 100μM AzA on +Zn or –Zn medium. Letters indicate significantly different values at p <0.05 determined by ANOVA and Tukey HSD. Multi-factorial ANOVA was used to test the impact of Genotype (Col-0, Sq-1 and *azi1* (*azi1-1*, *azi1-2*)), treatment (+Zn, -Zn, +AzA, -AzA) and their interaction on primary root length. The ANOVA results are presented in the table. Significative codes: ‘***’ 0.001 ‘**’ 0.01.

While *AZI1* had not been implicated in any known process involving Zn, it is known to mediate signal mobilization for systemic defence priming that can be triggered by AzA [16, 17]. We therefore hypothesized that *AZI1* would modulate growth and immunity programs depending on Zn and AzA status. To test this hypothesis, we first established the effects of the exogenous application of AzA on root growth. AzA affected root growth in a dose-dependent manner starting with a relatively mild reduction of growth at 100 μM to complete inhibition of root growth at 200 μM AzA (Fig 4A). We then determined whether this response is dependent on *AZI1*, and assessed root growth in Col-0, the Sq-1 accession and *azi1* mutant lines at 100 μM AzA and in presence or absence of Zn after 5 d of treatment. While, AzA severely inhibited root growth in Col-0 plants in presence of AzA and Zn (+AzA+Zn), the *azi1* mutant lines and the Sq-1 plants were significantly more resistant to the inhibitory effect of AzA (Fig 4B). This demonstrated that AzA modulates root growth in an *AZI1* dependent manner.

To test whether Zn modulates this response, we conducted the same assays under –Zn conditions. Strikingly, low levels of Zn alleviated the growth inhibitory effect of AzA on Col-0 to a large extent, and led to a further increased growth of the *azi1* mutant lines (Fig 4B). The primary root length of these mutant lines (*azi1*) grown in presence or absence of Zn and/or AzA was similar to those observed for Sq-1 genotype grown under same conditions (Fig 4B). Taken together, these data show that AzA induced reduction of root length is modulated by Zn levels, and that *AZI1* is a key component for this modulation. To further test whether the interaction between Zn and AzA is specific to early developmental stages or if it is retained later in plant development, Arabidopsis Col-0 and *azi1* mutant were grown for 10 days in +Zn condition, then transferred in +Zn, -Zn, +Zn+AzA, or -Zn+AzA conditions for 5 additional days. Also at this later stage, –Zn treatment leads to slightly increased primary root length of Col-0 while the root length of *azi1* is decreased compared +Zn treatments (S13A and S13B Fig). However, AzA application has an inhibitory effect on root growth regardless of the presence or absence of Zn in the medium in both Col-0 and *azi1* seedlings (S13A and S13B Fig). We therefore conclude that Arabidopsis plants could prioritize root growth over defence during early development in response to low Zn, which is in line with our initial GWAS analysis where the association of *AZI1* to primary root length in -Zn was highly significant during early development (day2) of Arabidopsis.

It is proposed that AZI1/AzA regulates one branch of SAR, and that the second branch is regulated by salicylic acid (SA) (for review, [29]). To determine whether the interaction of Zn limitation and AzA is specifically regulated by *AZI1*, or could involve SA-defence related genes, we assessed the effect of AzA treatment in presence or absence of Zn on primary root elongation in mutants for genes such as *ISOCHORISMATE SYNTHASE 1* (*ICS1*) and *CAM-BINDING PROTEIN 60-LIKE g* (*CBP60g*) (for review, [30]). Our results showed that the primary root lengths of 5-day-old *ics1* and *cbp60g* seedlings were similar to WT (Col-0) in presence or absence of Zn or AzA (S14 Fig). Therefore, we concluded that *AZI1* plays a specific role in regulating root growth in response to Zn limitation and in combination with AzA.

### Zn status strongly impacts immunity and modulates the response to AzA

**Fig 5:**
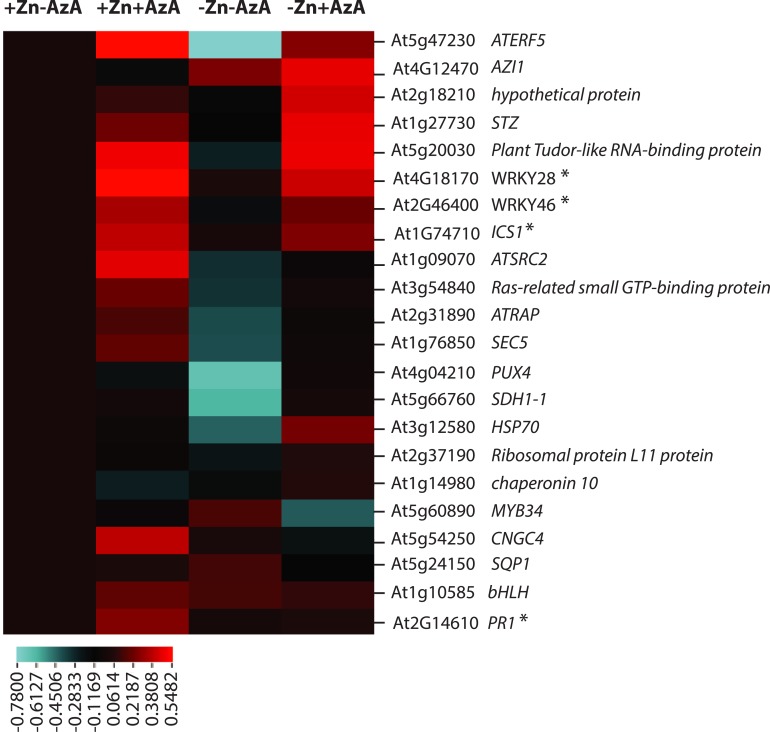
Expression levels of defence-related genes. Transcripts accumulation of At2g31890, At5g54250, At5g24150, At1g09070, At5g60890, At1g76850, At4g04210, At3g54840, At5g66760, At2g37190, At1g14980, At1g10585, *WRKY46* (At2g46400), At3g12580, *AZI1* (At4g12470), *ICS1* (At1g74710), At5g47230, *WRKY28* (At4g18170), At2g18210, At1g27730, At5g20030 and *PR1* (AT2G14610) in the roots *Arabidopsis thaliana* Col-0 genotype grown in +Zn or –Zn conditions with or without 100μM AzA. The Arabidopsis *Ubiquitin* gene was used as an internal reference. Experiments were independently repeated three times. Data was normalized to +Zn/-AzA condition, log2 transformed and visualized using "clustered correlation" CIM (http://discover.nci.nih.gov) [64]. Stars indicate genes involved in the biosynthesis and response to salicylic acid.

Our data had not only shown that Zn levels and AzA modulate root growth, but also that the root growth responses to these treatments strongly interact (Fig 4B). To test whether this interaction is due to the modulation of molecular responses to AzA by Zn levels, we measured the expression levels of 18 defence-related genes that had been shown a mild but significant expression change upon AzA treatment (P < 0.05) in leaves of wild-type plants (Col-0) [16], as well as 2 additional genes regulating salicylic acid biosynthesis (*WRKY28* and *WRKY46)* [31], [32, 33], *AZI1*, and a marker gene frequently used as a reliable molecular marker for SA-dependent SAR (*PR1*) [18]. Our q-RTPCR based gene expression analysis showed that almost all (16) of these defence-related genes were upregulated in response to the application of AzA (Fig 5). Notably, the group of most strongly induced genes contained genes involved in salicylic acid (SA) biosynthesis, such as *ISOCHORISMATE SYNTHASE 1*, *WRKY28*, *WRKY46*, as well as the SA response marker *PR1*. AzA treatment of plants grown on –Zn medium (-Zn+AzA) resulted in the upregulation of only 9 of the 16 genes that were upregulated in +Zn/+AzA (Fig 5). Notably, the SA response marker gene *PR1* was not among these. Furthermore, consistent with an effect of Zn levels on the expression of these genes, plants grown on low Zn showed a down-regulation of 6 of the 16 defence-related genes induced by AzA alone (Fig 5). Overall this suggests that interaction of –Zn and AzA is not due to a general lack of induction of SA biosynthesis genes, but rather acts more downstream during SA signalling. In roots of the Sq-1 accession (short root under Zn limitation) expression patterns provided a more complex picture (S7 Table). Here, the expression of *AZI1* was downregulated in response to -Zn-AzA and no significant changes were recorded for its expression in response to -Zn+AzA or +Zn+AzA treatments compared to control (+Zn-AzA). Expression of the *ICS1*, *WRKY28* and *PR1* genes showed an increase in response to -Zn-AzA, but no significant changes in response to -Zn+AzA compared to control (+Zn-AzA). Prompted by this difference to the Col-0 genotype, we measured whether the expression of Zn deficiency responsive genes (*ZIP3*, *ZIP5*, *ZIP12* and *PHO1;3*) in response to low Zn is altered upon AzA treatment in the Sq-1 accession. While expression of these four genes was induced by low Zn treatment (consistent with Sq-1 sensing the -Zn conditions), -Zn + AzA treatment significantly reduced the expression level of the four genes compared to low Zn treatment alone, AzA supply in Zn sufficient conditions significantly induced two of them (*ZIP3*, *ZIP5*) (S7 Table). Overall, these results demonstrate the presence of complex low Zn and AzA signal interaction in plants, and that Zn status impacts the expression of defence-related genes and modulates the response to AzA in plants, and that this is subject to natural variation.

### *A*. *brasilense* inhibits root growth in Arabidopsis in a Zn-dependant manner

**Fig 6:**
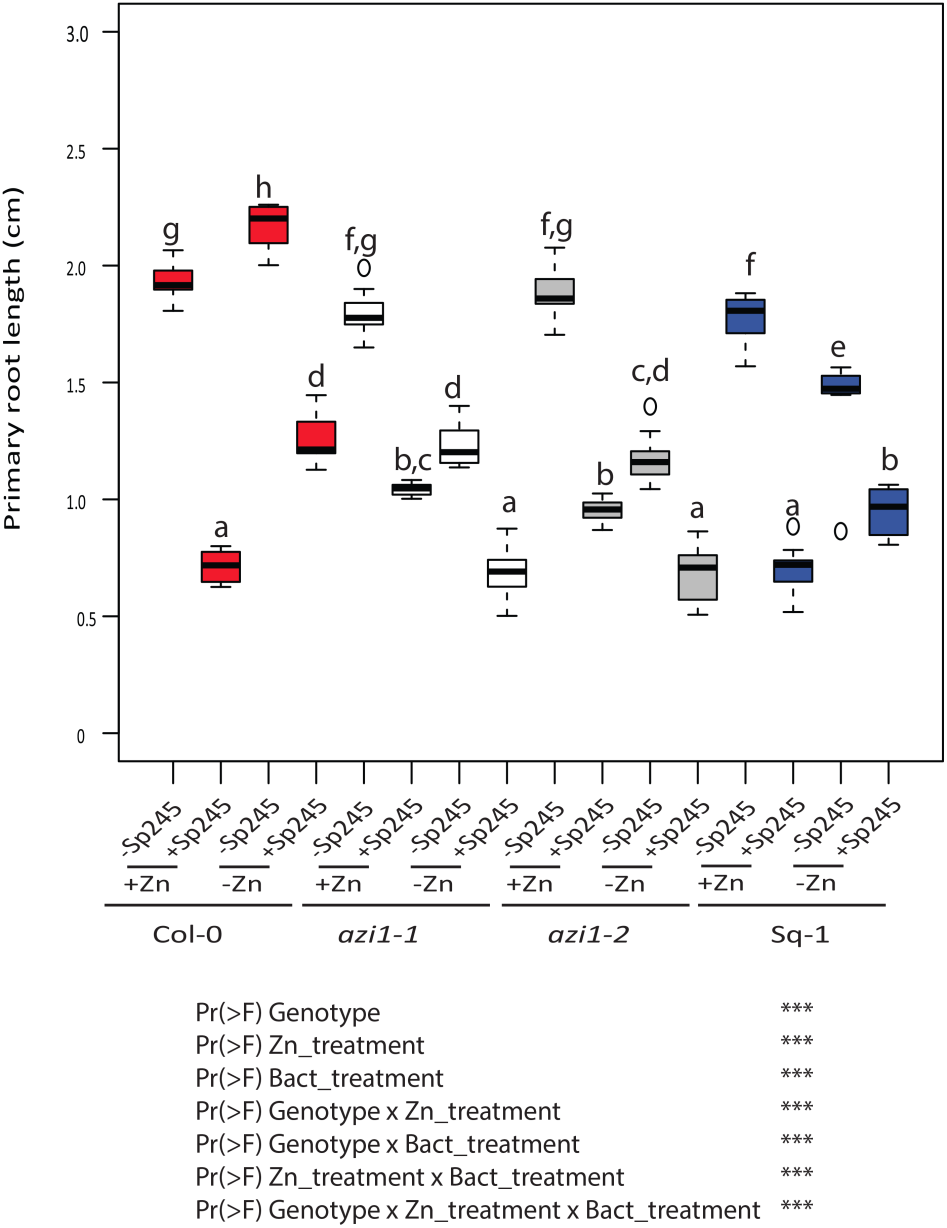
Zinc level and bacterial presence interact to regulate root length in an *AZI1* dependent fashion. Root lengths of Col-0, Sq-1, *azi1-1* and *azi1-2* seedlings grown with *Azospirillum brasilense* (Sp245) on +Zn or –Zn medium for five days. Letters indicate significantly different values at p <0.05 determined by ANOVA and Tukey HSD. Multi-factorial ANOVA was used to test the impact of genotype (Col-0, Sq-1 and *azi1* (*azi1-1*, *azi1-2*)), treatment (+Zn, -Zn, +BactSp245, -BactSp25) and their interaction on primary root length. The ANOVA results are presented in the table. Significative codes: ‘***’ 0.001.

The link of Zn status and *AZI1* to both, growth and defence prompted us to test whether interaction of *AZI1* and Zn status impacts growth responses to biotic stimuli. For this, we chose to measure primary root responses upon infection with the bacterium *Azospirillum brasilense* (Sp245) as it had been described that root growth as well as *AZI1* expression is changed in *A*. *thaliana* in presence of *A*. *brasilense* [34]. Consistently with [34] root growth of 5 d old Col-0 or Sq-1 plants was reduced when grown on complete medium (+Zn) and inoculated with *A*. *brasilense* (Fig 6). When grown on –Zn, this *A*. *brasilense* induced root length reduction was slightly less pronounced (Fig 6). The bacterially induced growth inhibition under +Zn was significantly less pronounced in the *azi1* mutant lines, showing that *AZI1* is involved in regulating this growth response. Under –Zn, bacterial incubation resulted in further decrease of root growth of *azi1* mutant lines compared to +Zn (Fig 6). Overall, these data clearly show that there is a complex interaction of Zn levels and that the *AZI1* gene determines the balance of growth and defence.

### Zn and AzA interaction is conserved in *Oryza sativa* root

**Fig 7:**
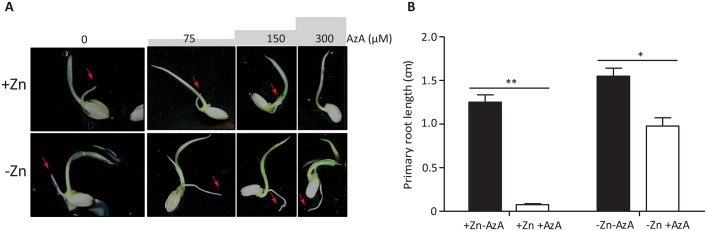
Zinc and azelaic acid interact to regulate root growth in *Oryza sativa*. A) Images of representative Rice (*Oryza sativa*, Nipponbare) seedlings grown on Yoshida medium supplemented with different AzA concentrations in presence or absence of Zn for five days. Red arrow indicates root. (B) Quantified primary root length of Rice seedlings (n = 10) in presence and absence of AzA and/or Zn. The data are given as means ± s.d. Asterisk indicates statistical significance, P < 0.05. Double asterisk indicates statistical significance, P < 0.01.

The gene family to which *AZI1* belongs, is strongly conserved throughout the *Viridiplantae* (green plants) [35]. This led us to hypothesize that the AzA mediated root growth regulation and its modulation by Zn status is a conserved growth-immunity regulating pathway. We therefore investigated the effect of AzA on root growth in presence and absence of Zn in the monocot species rice (*Oryza sativa*) (Fig 7A and 7B). Root growth increases in rice grown in low Zn compared to +Zn condition (Fig 7A and 7B). Interestingly, while seedlings grown in medium that contained AzA (300 μM) and Zn didn’t develop any roots, this growth inhibition was not observed when germinating the grains on –Zn medium containing AzA (Fig 7, S15 Fig). This demonstrated that AzA mediated growth inhibition, as well as its regulation by Zn levels is an evolutionary conserved mechanism.

## Discussion

Plants must sense changes in external and internal mineral nutrient concentrations and adjust growth to match resource availability [36, 37]. Responses to nutrient limitations manifest very early in plants life cycle, and root related processes are a major target of responses to nutrient constraints in plants. Consequently, primary root growth responds early on and drastically to nutrient limitations [38] and genome wide association mapping approaches are now being used to understand the genetic and molecular factors that govern these early growth responses [39]. However, apart from these abiotic variables (e.g. nutrients and water levels), the root is continuously exposed to changing biotic factors. Our study to identify genes and their variants that determine early root growth responses to low Zn levels revealed a genetic and molecular link between root growth response to abiotic (Zn) and biotic (defence related signalling) factors. In particular, our study revealed a significant association between the *AZI1* locus and primary root length in Arabidopsis accessions grown in –Zn condition, as well as an intriguing *AZI1* dependent interaction between Zn levels and the AzA pathway. One interesting question is whether the *AZI1* function that relates to abiotic factors is specific to Zn or not. While there has been one report in which it was shown that ectopic expression of *AZI1* improved Col-0 seed germination under high salinity stress condition and *azi1* mutants were overly sensitive [14], many leads point in the direction of a largely Zn specific function: Significant associations close to the *AZI1* locus were neither found in the GWAS for accessions that were grown at the same time on +Zn (S5 Fig), nor in other published root GWAS datasets for growth on MS medium [40], under different nitrogen conditions [41], under NaCl stress [42], and under Fe deficiency [39]. Moreover, while loss of function of *AZI1* caused decreased root growth of Arabidopsis plants grown under –Zn conditions, and *AZI1* overexpression caused the roots to be longer than WT (Fig 1), this was not observed when *azi1* or *35S*:*AZI1* were grown under -Fe conditions (S7C Fig). Nevertheless, the level of exclusiveness or the extent of this specificity of *AZI1* function for Zn can only be elucidated by further studies.

The responses that we have observed and studied occur early-on during plant development, when Zn levels in the seedlings will not be significantly depleted. Moreover, it is likely that traces of Zn will have remained in the washed agar that we used for these assays, generating an environment very low in Zn, rather than a fully Zn depleted growth environment. Overall, our findings thus exposed mechanisms that will relate more to sensing than to a Zn starvation/depletion response, consistent with our observation that these low Zn levels promote early root growth rather than to inhibit it. While we have identified a signalling mechanism that contributes to this –Zn dependent increase in root growth, the Zn sensor still remains elusive.

Our study proposes a possible mechanism for the regulation of root growth depending on the environmental Zn level, in which AZI1 plays an important role, and probably in an interaction with SA. AZI1- and SA-related signals are known to interact [17], and possibly involved in a self-amplifying feedback loop (for review [43, 44]). Our gene expression analysis revealed that Zn status impacts the expression of *AZI1*, as well as other immune- and SA-related genes in Arabidopsis (Fig 5). Variation in SA concentration in leaves and roots of Arabidopsis plants upon nutrient deficiency stresses has been reported [45], and appear to be nutrient specific [45]. For example, SA levels significantly increased in response to potassium deficiency while low Fe caused a significant decrease of SA in roots [45]. The involvement of phytohormones in controlling root growth under different nutritional status has been documented [46], and hormone accumulation “thresholds” appear to be critical for hormones action [47]. For example, in various plants species, treatment with low concentrations of SA led to root growth promotion while treatment with high concentration of SA caused an inhibition of root growth [43, 44]. In our study, -Zn treatment is associated with longer primary roots of wild-type plants (Col-0), together with no significant accumulation of SA as revealed by the absence of induction of a marker gene frequently used for SA accumulation, namely *PR1* [18]. Interestingly, in contrast to *azi1*, low SA-accumulating mutants, *ics1* and *cbp60g*, did show longer roots in –Zn similar to wild-type plants (Col-0). This suggests that the elongation of primary root in –Zn is a result of an active AZI1 and likely low SA level. The increase of SA [17], *AZI1* expression level and AzA [34] was reported in plants exposed to bacterial infection presumably to promote defence response, often at the cost of growth. In our gene expression analysis *PR1* was induced by +Zn+AzA conditions, suggesting an accumulation of SA under these conditions. Consistently, root growth of wild-type plants (Col-0) treated with (+Zn+AzA) was severely reduced compared to control condition (+Zn-AzA). Similarly, plants (Col-0) treated with Sp245 showed shorter root than the ones grown on control condition. The negative effect of either AzA or Sp245 treatment on root growth was alleviated when these stresses were combined with low Zn treatment, (-Zn+AzA or -Zn+Sp245). This was accompanied by an absence of *PR1* induction, indicating that the otherwise root growth inhibitory SA response is affected by low Zn levels at the molecular level. Our work exposed an interaction of Zn levels and immunity and a common genetic and molecular basis for this. Interestingly it seems that Arabidopsis can prioritize root growth over defence responses, if Zn levels are low during early development. Prioritizing root growth over defence in –Zn in early developmental stages could be a way to explore soil for available Zn. The data from rice point in the same direction and provide a hint that this interaction is evolutionary conserved. It will be interesting to elucidate how this relates to the molecular role of Zn in nutritional immunity in plants, perhaps somehow similar to the role of Zn levels in infection sites in animal systems [5–7].

*AZI1* belongs to a large family of pathogenesis-related proteins; LTPs. While some LTPs were suggested to play a role in defence reaction in a root specific manner [48], the role for AZI1 in defence responses was mainly investigated in aboveground tissues, and similar data for role of *AZI1* in roots are still absent. Our data demonstrate that the expression of AZI1 predominantly in roots is sufficient to control the primary root length in response to Zn availability. Much like *AZI1* function, the role of AzA had been also analysed only in the aboveground tissues. Our data reveal an important role of this pathway in the root, which extends the current models for underground defence priming. Importantly, the conserved specific interaction of Zn and AzA that can be observed not only in the dicot species Arabidopsis but also in the monocot species rice, is not only interesting from the view of basic biology, but also harbours very interesting perspectives for an innovative biotechnological application. This is because AzA is thought to directly mediate crop plant responses to pathogens and herbivores or to mimic compounds that do [49] and is listed among the natural compounds that induce resistance by a priming mechanism [50]. To activate these plant responses, AzA like others organic and inorganic chemicals can be applied as a foliar spray, seed treatment, or soil drench [49]. However, our work revealed that the soil-based application of AzA might significantly impact root traits depending on Zn bioavailability (AzA severely inhibited the root growth in +Zn), which could have an enormous and direct impact on plant growth in the field.

## Materials and methods

### Plant materials and growth conditions

We used 231 different genotypes of *A*. *thaliana* from different geographic origins (S1 and S2 Tables, S1A Fig) and for each genotype grew 12 seedlings. The previously described [51] *azi1* insertion mutant lines (SALK_017709 (N517709) and SALK_085727C (N657248) available in the Nottingham Arabidopsis Stock Centre. SALK_085727C was provided by Peter Urwin (University of Leeds, UK). The *ics1* (SALK_133146C) and *cbp60g* (SALK_023199C) mutants were used in this work. Lines overexpressing *AZI1* under the 35S promoter, under the *ZIP1* promoter, expressing the Col-0 or Sq-1 *AZI1* locus under their native promoters were generated in this *azi1* mutant background. Plant phenotyping for GWAS was as described previously [40]. Briefly, for each growth condition all lines were grown side by side. For both Zn conditions, GWAS assays were performed in the same growth chambers under the same 22°C long-day conditions (16 h light, 8 h dark). Seeds were placed for 1 h in opened 1.5 ml Eppendorf tubes in a sealed box containing chlorine gas generated from 130 ml of 10% sodium hypochlorite and 3.5 ml of 37% hydrochloric acid. For stratification, seeds were imbibed in water and stratified in the dark at 4 °C for 3 days to promote uniform germination. On each plate, eight different accessions with three seeds per accession were then germinated and grown in a vertical position on agar-solidified medium contained 0.5 mM KNO_3_, 1 mM MgSO_4_, 1 mM KH_2_PO_4_, 0.25 mM Ca(NO_3_)_2_, l00 μM NaFeEDTA, 30 μM H_3_BO_3_, l0 μM MnCl_2_, l μM CuCl_2_, 15 μM ZnSO4, 0.1 μM (NH4)6Mo7O24, and 50 μM KCl, in presence of 1% (wt/vol) sucrose and 0.8% (wt/vol) agar. -Zn or -Fe medium was made by not adding the only source of Zn (ZnSO_4_) or FeEDTA to the medium, respectively. For the assays involving azelaic acid treatments, AzA (246379 ALDRICH, Sigma) was added in different concentration ranging from 25 to 200 μM. For the assay with rice (*Oryza sativa* L.), Niponbare was used and seeds were soaked in deionized water over night in dark then transferred in a controlled-environment chamber (light/dark cycle of 14/10 h, 200 μmol photons m^-2^s^-1^, temperature of 28/25 °C and RH of 80%) to ¼ Yoshida media for 5 d [52, 53]: 0.36 mM NH_4_NO_3_; 0.41 mM MgSO_4_; 0.19 mM CaCl_2_; 0.13 mM K_2_SO_4_; 0.08 mM NaH_2_PO_4_; 4.72 μM H_3_BO_3_; 2.37 μM MnCl_2_; 8.90 μM Fe-NaEDTA; 0.62 μM ZnSO_4_; 0.04 μM CuSO_4_; 0.02 μM (NH_4_)_6_Mo_7_O_24_, adjust to pH 5.5. ZnSO4 was removed in -Zn medium. For rice treatments, azelaic acid (246379 ALDRICH, Sigma) was added in different concentration ranging from 75 to 300 μM. For the control condition, rice plants were kept in nutrient solution with the above-mentioned composition. Rice seedlings were grown in a growth chamber under the following environmental conditions: light/dark cycle of 14/10 h, temperature of 28/25 °C, and RH of 80%.

GWAS root trait quantification was conducted using the BRAT software [40]. Root length in assays involving mutant and transgenic lines, as well as rice plants was measured using ImageJ software, version 2.0.0 (http://rsb.info.nih.gov/ij/). Statistical differences between genotypes were calculated using t-test analyses and ANOVA with subsequent post hoc tests using Graphpad Prism (GraphPad Software Inc., San Diego, CA, USA) or Microsoft Excel (Microsoft, USA).

### Gene expression analysis by quantitative RT-PCR

Total RNA was extracted from roots of Arabidopsis wild type plants (different accessions) grown in presence or absence of Zn, and with or without azelaic acid (246379 ALDRICH, Sigma), using Plant RNeasy extraction kit (Qiagen) and RQ1 RNAse-free DNAse (Promega). Two μg of total RNA were used to synthesize cDNA using poly-A oligos. Real-time quantitative reverse-transcription PCR (RT-qPCR) was performed with a Light Cycler 480 Real-Time PCR System (Roche; Roche Diagnostics, Basel, Switzerland) using LightCycler 480 SYBR Green I Master mix (Roche, IN, USA). Primer list is provided in S8 Table. Primers were designed in conserved regions between tested accessions. Gene transcript accumulation quantification were performed in a final volume of 20 μL containing optimal primer concentration 0,3 μmol, 10 μL of the SYBR Green I master mix, and 5 μL of a 1:25 cDNA dilution. Real time-PCR conditions were as 95°C for 5 min, and followed by 40 cycles of 95°C for 10 s, 60°C for 10 s, 72 °C for 25 s, and finally one cycle 72 °C for 5 min. As a negative control, template cDNA was replaced by water. All PCR reactions were performed in triplicates. For each sample, a cycle threshold (Ct) value was calculated from the amplification curves. For each gene, the relative amount of calculated mRNA was normalized to the level of the control gene *ubiquitin10* mRNA (*UBQ10*: At4g05320). For every sample, the relative gene expression of each genes was expressed following normalization against the CT values obtained for the gene used for standardization, for instance ΔCT,*AZI1* = CT,*AZI1* − (CT,*UBQ10*). Quantification of the relative transcript levels was performed as described previously [54–57]. Briefly, -Zn treatment was compared to +Zn, the relative expression of a each gene was expressed as a ΔΔCt value calculated as follows: ΔΔCt = ΔCT,*AZI1*(-Zn) − ΔCT, *AZI1*(+Zn). The fold change in relative gene expression was determined as 2−ΔΔCT.

### Bacterial strains and growth conditions

Wild-type strain of *Azospirillum brasilense* is used in this study [58]. These bacteria strains were cultivated and inoculated in plant culture medium as described previously [59]. Statistical differences between genotypes were calculated using t-test analyses and ANOVA with subsequent post hoc tests using Graphpad Prism (GraphPad Software Inc., San Diego, CA, USA).

### Plasmid construction and plant transformation

The *AZI1* locus from Col-0 and Sq-1 accessions were cloned with primers spanning the region ranging from 1614 bp upstream of the *AZI1* transcription start site to the stop codon of *AZI1* into the binary vector pCAMBIA1301 by restriction enzymes of *BamHI* and *PstI* using primers listed in S8 Table. *AZI1* transcription start site to the stop codon of were cloned in fusion to 1129 bp *ZIP1* promoter using primers listed in S8 Table. The constructs were transformed into *Agrobacterium tumefaciens* strain GV3101 and then used for Arabidopsis transformation by the floral dip method [60]. Transgenic plants were selected by antibiotic resistance, and only homozygous descendants of heterozygous T2 plants segregating 1:3 for antibiotic sensitivity: resistance were used for analysis.

### GWA mapping

For GWAS, mean total root length values of 231 natural accessions were used (S1 and S2 Tables). The GWA analysis was performed in the GWAPP web interface using the mixed model algorithm (AMM) that accounts for population structure [23] and using the SNP data from the RegMap panel [61, 62] [19]. Only SNPs with minor allele counts greater or equal to 10 were taken into account. The significance of SNP associations was determined at 10% FDR threshold computed by the Benjamini-Hochberg-Yekutieli method to correct for multiple testing [24].

### Variance component analysis

Using a Multi-trait mixed model ([63]) we decomposed the variance of the root length response under plus/minus Zn Conditions for each day into a genetic term, the G X E interaction and an environmental term. The respective variance parameters were estimated in a null model without single marker effects and a global Kinship estimated from all markers. The analysis has been performed in R (R Development Core Team 2008). The respective R scripts are available at https://github.com/arthurkorte/MTMM.

## Supporting information

S1 Table Primary root growth of 231 natural accessions of *Arabidopsis thaliana* grown on zinc sufficient (+Zn) medium over 7 days after germination. (XLSX)

S2 Table Primary root growth of 231 natural accessions of *Arabidopsis thaliana* grown on Zn limiting conditions (-Zn) over 7 days after germination. (XLSX)

S3 Table Broad sense heritabilities for growth under +Zn and –Zn conditions. (XLSX)

S4 Table Zn response heritability and variance decomposition. (XLSX)

S5 Table Primary root length of accessions measured at day2 and *AZI1* locus marker SNP-allele (Chr4 7400493) of all accessions used in this work. (XLSX)

S6 Table GWAS result table of all marker SNPs from 231 natural accessions of *Arabidopsis thaliana* grown on Zn limiting conditions (-Zn) 2 days after germination. (XLS)

S7 Table Transcript levels of defence-related genes in wild-type plants grown in presence and/or absence of zinc and azelaic acid. (PDF)

S8 Table List of primers used in this study. (XLSX)

S1 Fig Genetic diversity and genotype by -Zn dependent root growth responses. A) Genetic diversity of accessions used in this study. Plotted are the two major principal components (PCs) from [19]that infer continuous axes of genetic variation. B, C) Mean root lengths (pixels) of accessions grown in +Zn (x-axis) or -Zn (y-axis) growth conditions on the days for which significant GWAS signals were found. Day 2 (B); Day 7(C).
(EPS)

S2 Fig mRNA abundance of Zn-responsive genes *ZIP3*, *ZIP5*, *ZIP12* and *PHO1;H3* in roots of Col-0 plants exposed to different Zn availabilities. Transcript levels of *ZIP3* (At2g32270), *ZIP5* (At1g05300), *ZIP12* (At5g62160) and *PHO1;H3* (At1g14040) in roots of Arabidopsis (Col-0) seedlings grown on vertical agar plate in presence or absence of Zn (day 5) as determined by Real-time qPCR. Transcript levels of these genes are expressed relative to the average transcript abundance of *UBQ10* (At4g05320) that was used as an internal control, and relative to +Zn values that were set to 1. Every data point was obtained from the analysis of roots collected from a pool of six plants. Data presented are means of three biological replicates ± SE. Asterisks indicate statistically significant differences compared to the +Zn treatment for each gene (P <0.05).
(EPS)

S3 Fig Frequency distribution of mean primary root length of Arabidopsis accessions grown on -Zn. Histograms of the daily frequency distribution of mean primary root length of 231 Arabidopsis accessions under -Zn conditions over a time course of seven days.
(EPS)

S4 Fig GWASs of mean root length grown on -Zn. Manhattan Plots showing the genome-wide associations of mean root length in a set of 231 *A*. *thaliana* accessions grown in presence of zinc for seven days. The chromosomes are represented in different colours. The horizontal blue dash-dot line corresponds to a nominal 0.1 significance threshold after Benjamini–Hochberg–Yekutieli correction. The red box indicates the significant association.
(EPS)

S5 Fig GWASs of mean root length grown on +Zn. Manhattan Plots showing the genome-wide associations of mean root length in a set of 231 *A*. *thaliana* accessions grown under Zn limiting conditions for seven days. The chromosomes are represented in different colours. The horizontal blue dash-dot line corresponds to a nominal 0.1 significance threshold after Benjamini–Hochberg–Yekutieli correction.
(EPS)

S6 Fig Primary root length of *azi1* mutant, 35S *AZI1* (OE *AZI*), Col-0 plants grown on +Zn and -Zn. A, B) Average primary root length of wild-type plants (Col-0 genotype), *azi1* mutant and overexpressor line 35S::*AZI1* (OE *AZI1*) plants grown under +Zn (A) or –Zn (B) over a time course of seven days. Experiments were independently repeated three times, and data are represented as mean ± s.d. n = 10. Letters a, b and c indicate significantly different values at p <0.05 determined by one-way ANOVA and Tukey HSD. C) Expression changes (fold change) of *AZI1* in Col-0 and *azi1* expressing pZIP1::*AZI1* grown for 5 days in –Zn conditions compared to Col-0 and *azi1* expressing pZIP1::*AZI1* plants grown in +Zn conditions respectively. Every data point was obtained from the analysis of roots collected from a pool of ten plants. Error bars correspond to s.d.; three biological repeats. The *Ubiquitin* gene was used as an internal reference.
(EPS)

S7 Fig *AZI1* is not involved in control of root growth under Zn or Fe limited conditions. (A) Representative root growth phenotypes of seedlings (day 5) grown under +Zn or –Zn conditions. Shown are wild-type plants (Col-0 genotype), *azi1-1* and *azi1-2* mutants and overxpressor line (OE *AZI1*) 35S::*AZI1* plants. (B) Average primary root length of wild-type plants (Col-0 genotype), *azi1* mutant and overexpressor line (OE *AZI1*) 35S::*AZI1* plants, day 5, grown under +Zn or –Zn respectively. (C) Average primary root length of wild-type (Col-0), *azi1-1* and *azi1-2* mutants and overexpressor line 35S::*AZI1* (OE *AZI1*) plants, day 5, grown under +Fe or –Fe, respectively. Experiments were independently repeated three times, and data are represented as mean ± s.d. n = 10. Letters indicate significantly different values at p <0.05 determined by ANOVA and Tukey HSD.
(EPS)

S8 Fig Pattern of expression of *AZI1* in Arabidopsis organs. Signal intensities for *AZI1* gene expression was extracted from AtGenExpress developmental dataset (http://jsp.weigelworld.org/expviz/expviz.jsp).
(EPS)

S9 Fig *AZI1* expression in roots is sufficient to modulate roots growth under Zn limited conditions. (A) Transcript levels of *AZI1* in roots of two transgenic lines that express *AZI1* under the control of the promoter of the zinc transporter ZIP1 (pZIP1::*AZI1* #1 and pZIP1::*AZI1* #2) in *azi1-2* background. Seedlings were grown on vertical agar plate in presence or absence of Zn (day 5). Data determined by Real-time qPCR. Transcript levels of *AZI1* gene is expressed relative to the average transcript abundance of *UBQ10* (At4g05320) that was used as an internal control, and relative to +Zn values that were set to 1. Every data point was obtained from the analysis of roots and shoots collected from a pool of six plants. Experiments were independently repeated three times. ND, not detectable. (B) Representative root growth phenotypes of seedling (day 5) grown under –Zn conditions. Shown are wild-type plants (Col-0 genotype), *azi1-2* mutant and two transgenic lines that express AZI1 under the control of the promoter of the zinc transporter ZIP1 (pZIP1::AZI1 #1 and pZIP1::AZI1 #2) in *azi1-2* background. (C) Average primary root length of wild-type plants (Col-0 genotype), *azi1-2* mutant and two transgenic lines that express *AZI1* under the control of the promoter of the zinc transporter ZIP1 (pZIP1::AZI1 #1 and pZIP1::AZI1 #2) in *azi1-2* background, day 5, grown under +Zn or –Zn respectively. Experiments were independently repeated three times, and data are represented as mean ± s.d. n = 10. Letters indicate significantly different values at p <0.05 determined by ANOVA and Tukey HSD.
(EPS)

S10 Fig Polymorphism patterns around the *AZI1* locus in extreme accessions. Gene models and SNP polymorphisms among representative extreme accessions (4 accessions with short root phenotype in -Zn and 4 accessions with long root phenotype in -Zn) for the region surrounding approximately 32kb upstream and 13kb downstream genomic region of *AZI1* gene. (A) Amino acid changes around *AZI1* (At4g12470) locus. (B) SNPs around *AZI1* locus.
(EPS)

S11 Fig Natural allelic variation at the *AZI1* locus is not relevant for root length variation in Zn sufficient condition. Primary root length of *azi1* lines complemented with pAZI1:*AZI1* from either Col-0 (n = 44), Sq-1 (n = 44) or the empty vector (n = 27) on +Zn conditions for five days. For each genotype, three repeats each containing five independent T3 lines. Box plots show analysis of the primary root length of *azi1* lines complemented with pAZI1:*AZI* from either Col-0, Sq-1 or the empty vector on +Zn conditions. Center lines show the medians; box limits indicate the 25th and 75th percentiles as determined by R software; whiskers extend 1.5 times the interquartile range from the 25th and 75th percentiles.
(EPS)

S12 Fig Alignment of the promoters (A) and coding region (B) of Arabidopsis *AZI1* genes from Col-0 and Sq-1 accessions. Alignment of DNA sequence of cloned genomic regions was done using the MultAlign program (http://multalin.toulouse.inra.fr/multalin/).
(EPS)

S13 Fig Effect of zinc and Azelaic acid levels in older roots. (A) Representative root growth phenotypes of Col-0 and *azi1-2* seedlings grown for 10 days in +Zn condition, then transferred in +Zn, -Zn, +Zn+AzA, or -Zn+AzA conditions for 5 additional days. (B) Root lengths of 15-day-old seedlings were measured. Box and whisker plots were generated using Boxplot, with the box represents the 25th to 75th percentiles and the whiskers reaching to the lowest and highest values. Letters indicate significantly different values at p <0.05 determined by ANOVA and Tukey HSD. DAG, days after germination. DAT, days after transfer.
(PDF)

S14 Fig Effect of zinc and azelaic acid concentration on root length in Col-0 (WT), *ics1* and *cbp60g* mutants. Root lengths of wild-type (Col-0), *ics1* and *cbp60g* seedlings treated with 100μM AzA on +Zn or –Zn medium for five days. Boxplots were generated by the standard R boxplot function. Data are represented as mean ± s.d. (n = 10). Letters indicate significantly different values at p <0.05 determined by ANOVA and Tukey HSD.
(EPS)

S15 Fig Conservation of the Zn/AzA interaction in rice. Rice (*Oryza sativa*, Nipponbare) seedling were grown for five days starting the day of imbibition on Yoshida medium supplemented with 300 μM of AzA in presence or absence of Zn. Scans were taken from 5-days old seedlings. Red arrows indicate roots.
(EPS)
