# Supplementary material for: Natural allelic variation of the AZI1 gene controls root growth under zinc-limiting condition
Source: PLoS Genet. 2018 Apr 2;14(4):e1007304. doi: 10.1371/journal.pgen.1007304 (PMC5897037; doi:10.1371/journal.pgen.1007304)
Supplement: S7 Table — (PDF) [file pgen.1007304.s007.pdf]

Table S7. Transcript levels of defence-related genes in wild-type plants grown in presence and/or absence of zinc and azelaic acid.

Accession	Treatments[x]				
	Gene Id	+Zn-AzA	+Zn+AzA	-Zn-AzA	-Zn+AzA
Col_0	**At1g12470**	1	$0,70^{y,**}$±$0,10^{z}$	$1,76^{**}$±0,14	$2,96^{**}$±0,35
	**At2g18210**	1	$1,24^{**}$±0,03	$0,73^{*}$±0,31	$2,68^{**}$±0,24
	**At5g60890**	1	$0,84^{*}$±0,09	$1,39^{**}$±0,10	$0,37^{**}$±0,03
	**At1g76850**	1	$1,56^{**}$±0,32	$0,40^{**}$±0,02	0,90±0,16
	**At3g12580**	1	$0,86^{*}$±0,18	$0,36^{**}$±0,18	$1,71^{**}$±0,21
	**At2g31890**	1	$1,37^{**}$±0,01	$0,40^{**}$±0,11	$0,86^{**}$±0,07
	**At1g74710**	1	$2,45^{**}$±0,04	$0,99^{*}$±0,12	$1,81^{**}$±0,14
	**At1g10585**	1	$1,53^{**}$±0,13	$1,34^{*}$±0,41	$1,21^{**}$±0,15
	**At2g37190**	1	$0,82^{**}$±0,07	$0,60^{**}$±0,29	$1,10^{*}$±0,38
	**At5g54250**	1	$2,43^{**}$±0,27	1,00±0,09	$0,62^{**}$±0,10
	**At1g14980**	1	$0,55^{**}$±0,22	$0,69^{**}$±0,50	$1,13^{*}$±0,05
	**At5g66760**	1	0,94±0,28	$0,24^{**}$±0,13	0,96±0,05
	**At3g54840**	1	$1,62^{**}$±0,16	$0,47^{**}$±0,23	0,94±0,11
	**At1g09070**	1	$2,92^{**}$±0,39	$0,48^{**}$±0,21	0,83±0,47
	**At5g24150**	1	$0,77^{**}$±0,16	1,33±0,82	1,04±0,52
	**At5g47230**	1	$3,55^{**}$±0,39	$0,16^{**}$±0,15	$1,87^{*}$±0,04
	**At4g04210**	1	$0,64^{**}$±0,25	$0,21^{**}$±0,03	0,92±0,11
	**At5g20030**	1	$3,10^{**}$±0,03	$0,54^{**}$±0,31	$3,07^{**}$±0,53
	**At1g27730**	1	$1,64^{**}$±0,05	$0,77^{**}$±0,07	$2,97^{**}$±0,04
	**At4g18170**	1	$3,48^{**}$±0,15	1,06±0,12	$2,61^{**}$±0,11
	**At2g46400**	1	$2,19^{**}$±0,05	$0,66^{**}$±0,06	$1,63^{**}$±0,06
	**At2g14610**	1	$1,84^{**}$±0,04	1,06±0,01	0,96±0,07
	**At2g32270**	1	1,20±0,27	$2,94^{**}$±0,65	$1,34^{**}$±0,07
	**At1g05300**	1	$1,60^{**}$±0,18	$2,53^{**}$±0,51	$1,58^{**}$±0,22
	**At5g62160**	1	1,10±0,13	$2,83^{**}$±0,55	$1,36^{**}$±0,26
	**At1g14040**	1	$1,92^{**}$±0,09	$1,78^{**}$±0,01	$1,42^{**}$±0,35
Sq-1	**At4g12470**	1	0,98±0,18	$0,82^{*}$±0,12	1,06±0,18
	**At4g18170**	1	$1,35^{*}$±0,33	$1,30^{*}$±0,41	$0,80^{*}$±0,08
	**At2g46400**	1	$1,32^{*}$±0,24	1,04±0,13	1,16±0,09
	**At1g74710**	1	1,13±0,22	$1,26^{*}$±0,18	0,82±0,32
	**At2g14610**	1	1,13±0,35	$1,38^{*}$±0,19	0,85±0,29

[x]The treatments are described in "Materials and Methods."

[y]The $2^{-\Delta\Delta Ct}$ value expresses the mRNA abundance for the gene of interest in a different conditions relative to the average mRNA abundance for the same gene measured in the control treatment. A $2^{-\Delta\Delta Ct}$ value of 2 means that the mRNA is two times more abundant in plants submitted to the considered treatment than in control plants.

[d]Average ± sd ($n \geq 4$).

* $p<0.05$, ** $p<0.01$.
