# Supplementary figures and images for: Natural allelic variation of the AZI1 gene controls root growth under zinc-limiting condition
Source: PLoS Genet. 2018 Apr 2;14(4):e1007304. doi: 10.1371/journal.pgen.1007304 (PMC5897037; doi:10.1371/journal.pgen.1007304)

Supplementary Figure 13

A

**Col-0**

*azi1-2*

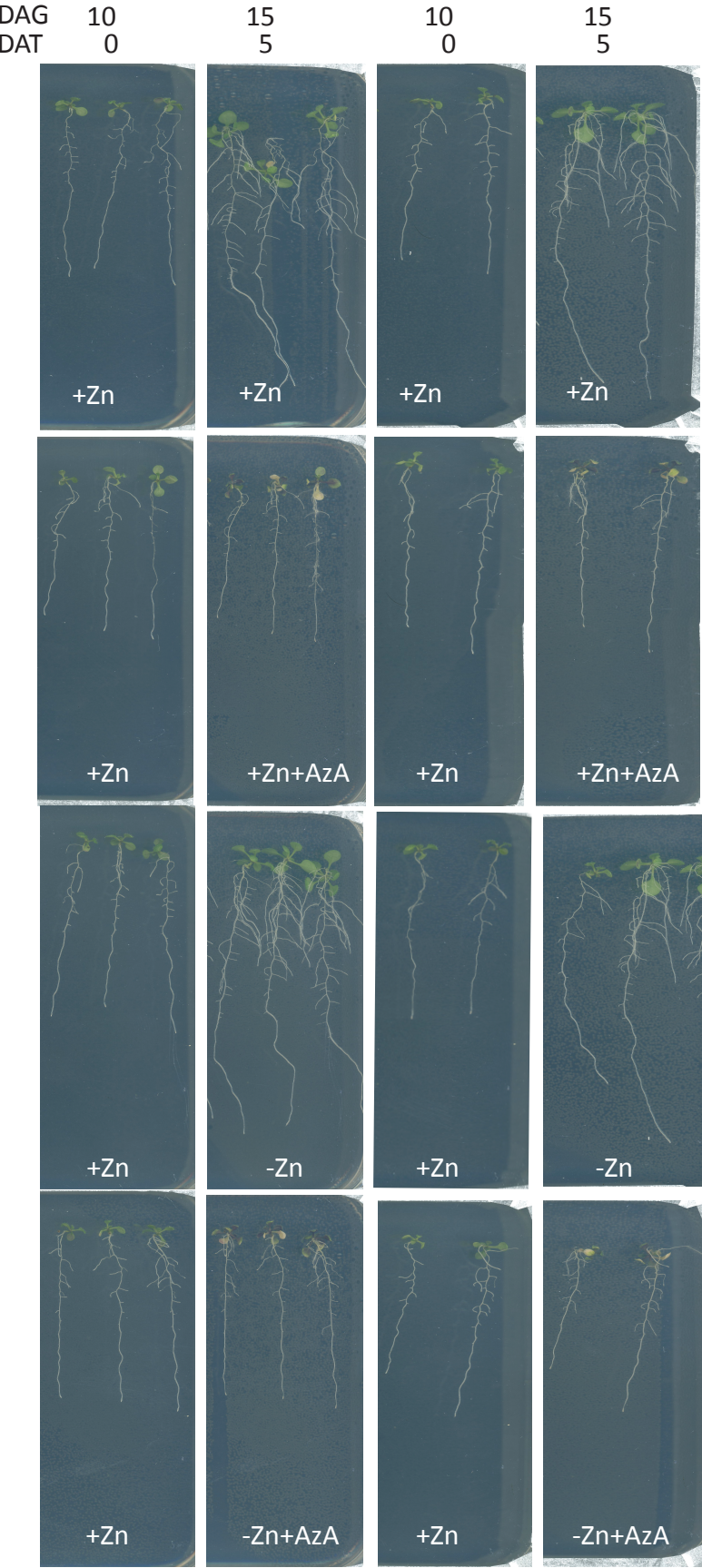

B

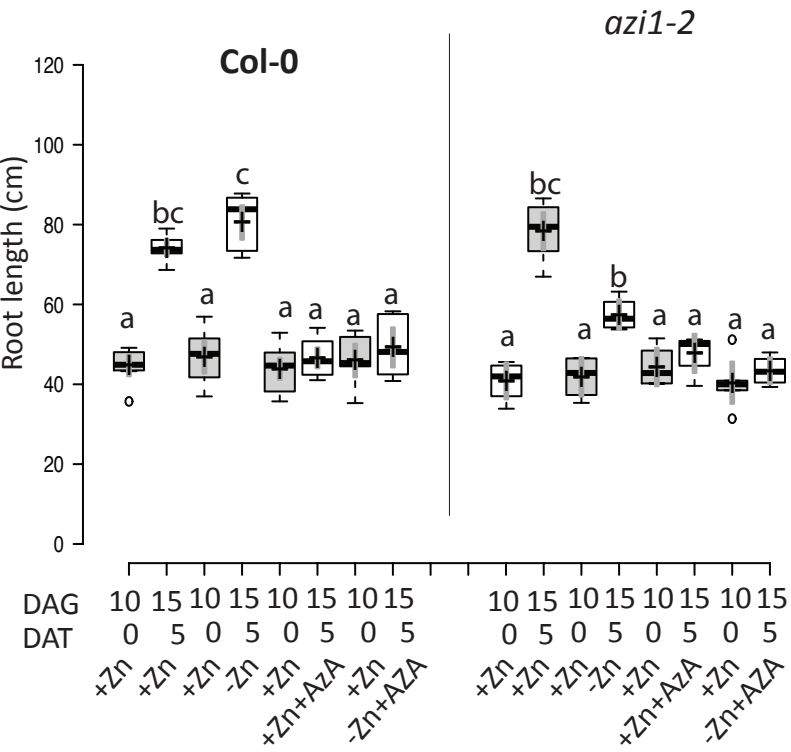

Supplement: S13 Fig — (A) Representative root growth phenotypes of Col-0 and azi1-2 seedlings grown for 10 days in +Zn condition, then transferred in +Zn, -Zn, +Zn+AzA, or -Zn+AzA conditions for 5 additional days. (B) Root lengths of 15-day-old seedlings were measured. Box and whisker plots were generated using Boxplot, with the box represents the 25th to 75th percentiles and the whiskers reaching to the lowest and highest values. Letters indicate significantly different values at p <0.05 determined by ANOVA and Tukey HSD. DAG, days after germination. DAT, days after transfer. (PDF) [file pgen.1007304.s021.pdf]
